# Considerations on staffing levels for a modern assisted reproductive laboratory

**DOI:** 10.5935/1518-0557.20220048

**Published:** 2023

**Authors:** Romualdo Sciorio, Raffaele Aiello, Ronny Janssens

**Affiliations:** 1 Edinburgh Assisted Conception Programme, EFREC, Royal Infirmary of Edinburgh, 51 Little France Crescent, Old Dalkeith Road, Edinburgh, Scotland, EH16 4SA, UK; 2 OMNIA Lab S.C.a.R.L, Via Cesare Rosaroll 24, 80139 Naples, Italy; 3 BE-ART IVF, Kloosterstraat 76, 2880 Bornem, Belgium

**Keywords:** medically assisted reproduction, embryo culture, embryology laboratory, human resourses, increased laboratory procedures, adequate staffing levels

## Abstract

The duties recently performed in the embryology laboratory have deeply increased compared to those realized a couple of decades ago. Currently, procedures include conventional in vitro fertilization (IVF) and ICSI techniques, or processing of surgically retrieved sperm, embryo culture and time-lapse monitoring, blastocyst culture, as well as trophectoderm biopsy for preimplantation genetic testing and cryopreservation. These techniques require not only time, but also high knowledge level and acutely concentration by the embryologist team. The existing data indicate that an IVF laboratory need to have adequate staffing levels to perform the required daily duties, and to work in optimal conditions that are critical to assure a high quality service, as well as avoiding incidents and to provide the best outcomes. As a result, IVF clinics have invested in human resources, but there is still a large discrepancy between IVF centres on the number of embryologists employed. Currently there is no golden standard on the human resource requirements for assisted reproductive technology procedures; therefore, in this review paper we aim to provide arguments to take into account to determine the embryology staffing requirements in an embryology laboratory to assure optimal safety and efficiency of operations.

## INTRODUCTION

**Figure 1:**
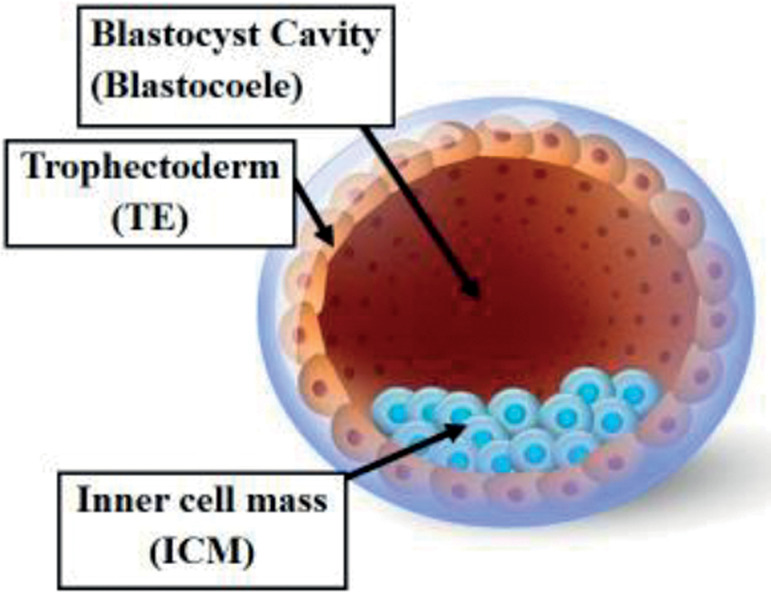
Five days after fertilization the human embryo forms the blastocyst, composed of two differentiated cell types and a central cavity filled with fluid (blastocoel cavity). The centrally located group of cells: the inner cell mass (ICM) will become the fetus and the surface cells that surround the cavity are called the trophectoderm (TE) and will later develop into the placenta.

Medically assisted reproduction (MAR) treatment is a high-complexity multi-step procedure, which has markedly evolved over the last decades (Thoma *et al*., 2013; De Geyter *et al.*, 2018). The complexity of MAR treatment has increased compared to an IVF cycle performed at the end of last century. The evolution of more physiological culture media, led to the generalised embryo culture to the blastocyst stage (Figure 1), aiming to enhance both uterine and embryonic synchronicity, and to obtain an increased pregnancy outcomes compared to that achieved with transfer of cleavage stage embryo (Gardner & Schoolcraft, 1999; De Vos *et al*., 2016). *In vitro* culture to the blastocyst stage implicates extra time, including media replacement on day-3 and embryo assessment at blastocyst stage. Further, preimplantation genetic testing, involves additional work to perform embryo biopsy, as well as communication with the genetic laboratory and patients. In addition, freezing human gametes and embryos have significantly enhanced, particularly due to the improved results obtained with the vitrification protocol, which recently has almost replaced the slow-freezing procedure previously used to cryopreserve human embryos/oocytes (Sciorio *et al*., 2018; 2019; Rienzi *et al*., 2017). Indeed, cryopreservation has taken an important role in assisted reproductive technology (ART) and it is applied to lower the occurrence of multiple pregnancies (Sullivan *et al*., 2012; Van Montfoort *et al*., 2005; Johnston *et al*., 2014) and to overcome the time interval between blastocyst biopsy and genetic result. Furthermore, in the last decades due to the equal opportunity for transgender individuals, MAR treatment are practiced for those couples as well as single women/men and homosexual couples (Mackenzie *et al*., 2020). Gamete donation program requires extra time and it implies a meticulous handling of data and matching, high skills in performing the oocyte or sperm warming process, with subsequent fertilization and embryo culture. Human embryogenesis demands a more critical growth environment as gametes and embryos are especially sensitive cell types, largely unprotected as they lack epithelial surfaces, thus needs to be treated by the embryology team with extremely care, attention and concentration. IVF laboratory with shortage of staff, working under pressure or tired due to too high workload will take shortcuts and hurry. These staffing issues are associated with loss of attention, reduced concentration leading to and might increased risk of committing errors or accidents with potentially severe consequences. Therefore, the goal of this opinion paper will be to illustrate the main principles of modern embryologist laboratory and the time associated for each treatment, which has changed extensively compared to a tradition IVF cycle performed few decades ago. Thus, we suggest here a proposal to estimating the embryology personnel required in a modern IVF laboratory, and we present a cogent method to determine minimum staffing levels to assure quality and safety.

## IVF CYCLE IN THE 1980S COMPARED TO THE MODERN TREATMENT

From the beginnings of IVF, embryos have been always selected for transfer based on their development and evaluated by non invasive approaches (Edwards *et al*., 1984; ESHRE Guideline Group on Good Practice in IVF Labs, 2016), wich have restricted and peculiar limitations especially due to the high inter-observer variability (Braude, 2013). The complexity of contemporary MAR practice has deeply changes compared to a tradition IVF cycle performed during the 1980s and 1990s. At that time, an ART cycle involved mainly standard IVF insemination (very few cases with ICSI insemination); embryos were being cultured until days 2 or 3 and transferred according to morphological evaluation based on the number and size of blastomeres, degree and pattern of fragmentation and multinucleation (Edwards *et al*., 1984; ESHRE Guideline Group on Good Practice in IVF Labs, 2016; Braude, 2013). The slow freezing protocol was rarely applied to freeze the supernumerary embryos after transfer. Modern ART have endorsed the introduction of preimplantation genetic testing for aneuploidy (PGT-A), previously called preimplantation genetic screening (PGS), and preimplantation genetic testing for monogenic diseases (PGT-M) also named preimplantation genetic diagnosis (PGD), and many IVF units have invested in adequate technologies and staff to provide those services. This practice was first proposed by Handyside *et al*. (1990). New advances in genetic and molecular screening have been applied to identify euploid embryos with more accuracy, and their replace should increase pregnancy outcome. PGT-A is recommended for: advanced maternal age (AMA), repeated implantation failure (RIF), and for patient with history of recurrent pregnancy loss (RPL). Practically, the *in vitro* embryo in the embryology laboratory is biopsied and screened for chromosomal anomalies prior to transfer into the woman uterus (Sciorio & Dattilo, 2020). The genetic screening is reliant to the blastocyst culture, which currently has become a routine practice in the embryology laboratory. This has been possible with the establishment of new culture media and reliable incubators, which can assure stable culture conditions (Sciorio & Smith, 2019).

## INSEMINATION TECHNIQUE (IVF-ICSI-IMSI)

Since the early day of IVF, the main procedure adopted to cure infertile couples was using the standard IVF insemination. At the time of oocyte retrieval, the cumulus-oocyte-complexes (COCs) were removed from follicular fluid and cultured in specific equilibrated culture media at 37°C and 6% CO_2_ in atmospheric air in incubator. Semen sample, produced by masturbation and processed to select the best motile sperm and then used for insemination (Bourne *et al*., 2004). Normal fertilization was established under microscope identification of the two pronuclei almost 16-18 hours post insemination. As far as time is concerned, the insemination process is very straight forward, and it need only a short time to release a specific amount of motile sperm into the dish containing culture media and COCs. However, it was soon evident that conventional IVF was much less effective in case of male factor infertility (Devroey & Van Steirteghem, 2004; Fishel *et al*., 2000). Therefore, since the early 1990s different techniques have been enforced in order to enhance fertilization and pregnancy outcomes for couples with severe male subfertility, including partial zona dissection (PZD) and of subzonal microinjection of spermatozoa into the perivitelline space (SUZI). In 1992, intra-cytoplasmatic sperm injection (ICSI) was reported by Palermo *et al*. (1992), where a single spermatozoon was injected into the oocyte cytoplasm. The ICSI technique represented a huge advancement, since complete fertilization failure was often reported with suboptimal sperm features and IVF insemination (Fan *et al*., 2012; Coates *et al*., 1992; Xie *et al*., 2013). Consequently, ICSI became quickly applied to treat patients with male infertility (Payne *et al*., 1994; Palermo *et al*., 1993). In addition, alternative technique for sperm selection were proposed, as the one described by Sakkas *et al*. (2015), who reported the utility of hyaluronic acid (HA) binding at the time of selection the motile sperm to inject. Further, another micromanipulation technique was described named intra-cytoplasmic morphologically selected sperm injection (IMSI) consisting in the injection into the oocyte of a sperm which was widely analyzed for morphological evaluation. With the introduction of IMSI, the embryologist by increasing the resolution of the optics, would be able to better assess the motile sperm in details, identifying vacuoles, as well as the shape or any other structural defects, and therefore was supposed to optimize the ICSI outcomes (Bartoov *et al*., 2003; Berkovitz *et al*., 2006). Subsequently, due to the high fertilization rate, the ICSI application increased worldwide, and it becomes applied to couples without male factor infertility. In Europe, in 2012 ICSI was used in about 70% of all IVF treatment compared to 35% in 1997. Some countries as Turkey, South-East Asia, Philippines, Middle East and South America ICSI is performed in 100% of IVF cycles (Kim *et al*., 2007). In the USA, between 1996 and 2012, the use of ICSI in MAR treatments has raised from 34% to 76% (Khamsi *et al*., 2000). Despite the extensive spread of ICSI in patients with non-male factor infertility, there is a little evidence on its effectiveness in this population in terms of pregnancy outcome (Kim *et al*., 2007; Plachot *et al*., 2002). Several studies have indicated that ICSI adopted in couple with non-male infertility might not improve the clinical outcomes (Tannus *et al*., 2017). Indeed, there are still some concerns about the ICSI safety, which generatee a strong debate. The main consideration is associated to the occurrence of epigenetic modifications and imprinting disorders in babies conceived following ICSI. There is some evidence showing a raised risk of imprinting disorder in babies conceived adopting MAR treatments compared to naturally conceived babies. However, those studies are still preliminary and further investigations urgently needed to confirm those results (Hiura *et al.,* 2014; Lazaraviciute *et al*., 2014; Vermeiden & Bernardus, 2013; Anckaert *et al*., 2013). Further concern exists on the unnecessary use of ICSI, which is correlated to a higher cost and might be considered unethical, as well as extra operator time consumed (Wen *et al*., 2012; Lie *et al*., 2005). Indeed, ICSI procedure is more labour-intensive and time-consuming compared to the standard IVF insemination, it requires in average the triple amount of time, depending on the number of oocytes to be injected and the quality of sperm (Alikani *et al*., 2014). Further, significant extra time is required to complete staff training, to allowing the acquisition of the right skills and knowledge to make an operator capable to perform ICSI technique.

## THE IMPACT OF CRYOPRESERVATION IN A MODERN ART LABORATORY

Cryopreservation technology has firmly established its leading role in a modern IVF laboratory. A massive progress in the field was obtained with the vitrification protocol, firstly applied in Japan and Australia (Kuwayama *et al*., 2005; Kuleshova *et al*., 1999). Vitrification was introduced as a novel method to cryopreserve human embryos, with the aim to provide higher success rates in terms of survival at the warming process and implantation potential (Sciorio *et al*., 2018; Rienzi *et al*., 2017; Sciorio *et al*., 2019). The vitrification program has resulted to be decisive in reducing the multiple pregnancy rate in ART treatments, and to increasing the application of single embryo transfer (Sullivan *et al*., 2012). In addition, vitrification has allowed the application of the “freeze-all” (FA) strategy or “elective frozen embryo transfer” (eFET), which involves the cryopreservation of all viable embryos to be transferred in subsequent cycles, thus avoiding the supra-physiologic hormonal levels observed during ovarian stimulation (OS). It is well reported that the occurrence of ovarian hyperstimulation syndrome (OHSS) during OS is one of the complications observed in the ART treatment, which is a potentially life alarming condition (Kawwass *et al.,* 2015). The first report illustrating the utility of the FA approach was published more than twenty years ago (Ferraretti *et al*., 1999), nowadays this strategy represents the golden standard in patients at high risk of OHSS (Dosouto *et al*., 2017; Sciorio & Esteves, 2020). Vitrification necessitates high technical skill and embryologist knowledge, and it is time consuming, especially if there are a large number of embryos or oocyte to vitrify. ART units should therefore provide additional staff training which is mandatory, before the vitrification process can be applied routinely. Indeed, the vitrification protocol requires intense precision from the operator. The oocyte or embryo is placed in the equilibration solution (for 8 to 12 minutes: depending on the protocol used), and then moved to the vitrification solution for only 45-60 seconds. The warming process needs to be completed with similar skills, respecting the time, in order to remove the cryoprotectant from the warmed cell(s), and replaced to the culture medium (Liebermann & Tucker, 2006). Indeed, it is important that ART centres have daily a specific number of trained staff allocated to performing the vitrification-warming procedures.

## PREIMPLANTATION GENETIC ASSESSMENT, TROPHECTODERM BIOPSY AND CRYOPRESERVATION

As mentioned earlier the introduction of PGT-A and PGT-M in modern ART laboratory intent to increase pregnancy outcomes following the transfer of euploid embryo. This approach has introduced a considerable difference in the daily embryology duties. Although the debate on the efficacy of the genetic screening is still ongoing and several studies have proposed queries on its efficiency (Mastenbroek *et al*., 2007; 2011; Scott *et al*., 2013; Sciorio & Dattilo, 2020), we want to highlight here the extra time needed to complete this practice. For safety, it needs to be performed under a strict human double witness, especially at the time of the embryo biopsy. Currently, the trophectoderm biopsy at the blastocyst stage is considered the golden standard to perform biopsy. In the early days of genetic screening, the biopsy was performed on the cleavage stage embryo, where one or two cells were removed from an eight-cell embryo and genetically analysed (Sciorio & Dattilo, 2020). Cleavage stage embryos might hold high levels of mosaicism, therefore to overcome this concern, a blastocyst stage biopsy was proposed, whereby 5 to 10 trophectoderm cells are removed from the embryo and assessed. This should provide an increased and more accurate detection of mosaicism (Scott *et al*., 2013). However, blastocyst culture and biopsy mean that the embryology labortory need to be ready to perform such technique on days 5 and 6 of culture, even during the weekend. Therefore, the embryology clinic should allocate at least two trained operators to perform biopsy, witness and vitrification. Recent study has noticed that even day-7 blastocysts, can achieve an acceptable level of quality, and following genetic assessment they have shown euploid rates comparable to day-6 blastocysts and resulting in healthy live births following frozen embryo replacements (Minasi *et al*., 2015; Whitney *et al*., 2016; Hammond *et al*., 2018). Additional work at the biopsy is related to the vitrification procedure, which has built a strong bond with PGT-A and PGT-M programs. Following blastocyst biopsy, the embryo needs to be vitrified in order to allow the genetic laboratory to overcome time restraints between biopsy and diagnosis. Once the results are obtained, the blastocyst needed to be warmed and replaced in a subsequent menstrual cycle. Human double witnessing at this stage is extremely important, additionally each blastocyst need to be vitrified in one device, in order to follow a specific identification code, which at the warming step will identify the euploid embryo to be replaced. Of course, training and continuing professional development (CPD) is necessary and all embryology staff should have an efficient system to maintain skills and knowledge up to date. In order to be competent for a specific task, such as micromanipulation techniques or vitrification, embryologist staff need to invest time in practising, and ideally, those sections should be recorded in a logbook. Once a specific number has been reached with optimal standard, the operator might be allowed to perform the duty indipendently (Alpha Scientists in Reproductive Medicine, 2015).

## THE OOCYTE DONATION PROGRAMME

The advantage of the vitrification has represented a clear breakthrough for oocyte cryopreservtion. The oocyte is a remarkably sensitive cell and it is difficult to freeze, mainly due to its large size and the high amount of water in the cytoplasm, which might generate intracellular ice and kill the cell (Paynter *et al*., 1999). One of the benefits of the vitrification relates to the optimal survival rate after the warming process and the acceptable pregnancy outcome following the replacement of embryo developed after oocytes warming, fertilization and *in vitro* culture (Cobo & Diaz, 2011; Cobo *et al*., 2014). Since 2013 when the ASRM removed the empirical logo (Practice Committees of the American Society for Reproductive Medicine and the Society for Assisted Reproductive Technology, 2013) the practice of oocyte cryopreservation is expanded a lot, and its clinical application has deeply increased in both social fertility preservation (FP) and for cancer patients (Sciorio & Anderson, 2020). In the last twenty years, we have witnessed an increase occurrences in female cancer disease. At the time of diagnosis, only a small percentage of young women are informed about becoming infertile following cancer treatments. Oncology has intensively grown and nowadays many drugs are available to block cancer advancements, however a side effect of those treatments might be associated to reduced reproductive function and gonadotoxic effect (Loren *et al*., 2013). Breast cancer for example is one of the most common cancer in women. It has been reported that more than 10% of new cases are diagnosed in women of reproductive age (Kim *et al*., 2016). In addition, with the social tendency of delaying motherhood until later in life, there are a raising number of women who have not completed parenthood at the time of cancer diagnosis. Therefore, considering that chemotherapy might induce premature ovarian insufficiency and infertility, the oocyte cryopreservation before cancer treatment represents a valid and established method to preserve their fertility and to obtain a healthy baby in the future (Sciorio & Anderson, 2020; Merlo *et al*., 2012; Meirow *et al*., 2010). The feasibility to successfully cryopreserve the oocyte has made the synchronization process in egg donation program between the donor and the recipient much easier. Indeed, it has been seen a deeply decrease in women’s fertility especially in those at advanced maternal age (Perheentupa & Huhtaniemi, 2009). There are several conditions affecting fertility potential, including premature ovarian failure, reduction in the ovarian follicular reservoir compromise oocyte quality. Therefore, the application of oocyte donation has become more common and is nowadays considered a well accepted procedure to manage untreatable female infertility (Sauer & Kavic, 2006). This approach was first applied in Australia by Trounson *et al*. (1983) and is nowadays well-established for age-related female infertility. The programme involves COCs retrieval from a donor, insemination with sperm from the recipient’s partner, fertilization, *in vitro* culture, and embryo transfer to the recipient’s uterine cavity. In case of logistical difficulties or lack of donors, oocytes can be collected and vitrified, stored in liquid nitrogen and carefully transported to another IVF unit, located in another part of the country or abroad (Alikani & Parmegiani, 2018). This led to the establishment of egg-banks for the use of vitrified-warmed donor oocytes, located abroad and shipped to the recipient region. This approach overcomes limitations linked to the lack of donors, which can be an issue in some country (Sciorio *et al*., 2021a; Rienzi *et al*., 2020) however, it necessitates extra time from the embryology team, high level of coordination and data sharing, including private and confidentially information transmitted between the centre shipping the gametes and the recipient unit. Important information needs to be exchanged among the embryologist teams of the units, such as the culture media used or the vitrification protocol applied for the cryopreservation. Extra time will be required for administration of cryopreserved gametes or embryo, including the maintaining an inventory and organising the import and export. In some units the embryologist team is also involved in the coordination between donor and recipient, this task implicates extra communication with patients, which require other time. Moreover, advancements in cryotank malfunction and troubleshooting are also imperative. Some cryogenic tank, containing cryopreserved gametes and embryos are equipped with alert systems feature a scale underneath to monitor weight changes and detect leakage of nitrogen, as well as shift in temperature. Finally, additionally time is necessary to remain up to date with regulations (Alikani *et al*., 2014; Practice Committees of the American Society for Reproductive Medicine and the Society for Assisted Reproductive Technology, 2013).

## FERTILITY OPPORTUNITY FOR TRANSGENDER PATIENTS

In the recent decades, fertility preservation has mainly been applied for social reasons and in cancer patients as described above. This field now represents a great opportunity to conserve future reproductive ability for transgender patients. Gender diversity involved the broad range of forms in which personal gender identification might contrast from the sex at birth, which might drive to physical and critical emotional distress (Gooren, 2011). It has been reported that transgenders have the same desire to get own babies as for cis-gender persons. Studies have found that more than 50% of transgender patients desire to have future children and among 37% to about 70% would consider FP (Wierckx *et al*., 2012). However, a large multicentre study published by Auer *et al*. (2018), conducted in Germany reported that only a small percentage of 9.6% of transwomen and about 3% of transmen had indeed experienced FP. A frequent path for transgenders is the adoption of hormonal therapy to mitigate gender dysphoria and live well with the desired gender. Although the physical changes associated with sex hormone are normally linked to a better mental well-being, but consequences are paid by the lost of future fertility (Hembree *et al*., 2017). The best option for FP in transwomen individuals is to cryopreserve semen samples before to start the hormonal therapy and oocyte or embryo cryopreservation for transmen after OS. Sperm cryopreservation and storage in nitrogen liquid is a well-established procedure. The semen can normally be produced by masturbation, which might be problematic for transwomen, especially if the hormonal therapy has already started resulting in increased difficulty for erection and ejaculation (De Roo *et al*., 2016). FP is a quickly evolving area of reproductive medicine, and supplying the right information to transgender facing the loss of fertility through hormone therapy is evolving to standard of care. Transgenders should be informed about the advantages in cryopreservation technique in order to achieve a pregnancy in the future; therefore, reproductive counselling is very important.

## EMBRYO CULTURE WITH TIME-LAPSE MONITORING AND ARTIFICIAL INTELLIGENCE

A considerable improvement in culture condition has been the introduction of a new type of incubators, with integrated time-lapse monitoring (TLM) technology and specifically designed to culture human embryos. This novel approach merges three elements: an incubator, a microscope and imaging software. The union of those components brings a constant embryo monitoring from early stage of fertilization to the blastocyst formation (Meseguer *et al*., 2012; Basile *et al.,* 2013; Aparicio-Ruiz *et al*., 2016; Sciorio *et al*., 2021b; Sciorio & Meseguer, 2021). In addition, it provides a steady an uninterrupted culture conditions and avoids the need to move embryos outside of the incubator exposing them to un-physiologic environment (Sciorio & Smith, 2019; Zhang *et al*., 2010). In the last decade, plenty of literature have shown the potential benefit of this technology, and some studies have correlated specific key timing parameters to blastocyst formation and pregnancy outcome (Meseguer *et al*., 2012; Basile *et al*., 2013; Aparicio-Ruiz *et al*., 2016; Wong *et al*., 2010; Sciorio *et al*., 2021b; Sciorio & Meseguer, 2021). Other aspects of embryo development have been described as poor-prognosis factors, such as direct, irregular or reverse cleavages or blastocyst collapse(s), and might be used as deselection criteria (Desai *et al*., 2018; Stecher *et al*., 2014; Sciorio *et al*., 2020a,b; Sciorio & Meseguer, 2021; Sciorio *et al*., 2021b; Sciorio *et al*., 2020a). Advances in TLM have generated the evolution of specific algorithms, based on computer process of a large amount of data and images, and try to establish a link with embryo viability and implantation potential. As well as very recently, artificial intelligence (AI) defined as the capacity of machines to learn and display intelligence, and machine learning (ML) based on the concept that higher-powered computer can learn to process data without human supervision. Those applications have been used by Khosravi *et al*. (2019) to predict blastocyst quality investigating more than 10.000 embryos. Similarly, Tran and collaborators in a retrospective analysis applied the deep learning model for automatically recording morphokinetic videos, and analysing more than 10.000 videos were able to recognize images of blastocysts that generated a foetal heartbeat (Tran *et al*., 2019). Although those are very preliminary studies, and further validation needs to clarify the benefit of this approach, it results very promising, and may be in the next couple of decades will be become routinely applied in ART laboratory to cooperate with the embryologists to the process of embryo selection. However, currently the process of annotation is still performed manually by an embryologist, and it needs a certain amount of time and further might be slightly operator-dependent. Finally, most of TLM are still quite expensive; necessitate significant training before it can be routinely used, as well as regular services and maintenance for the software updates.

## WITNESS PROCEDURE IN ART

**Figure 2:**
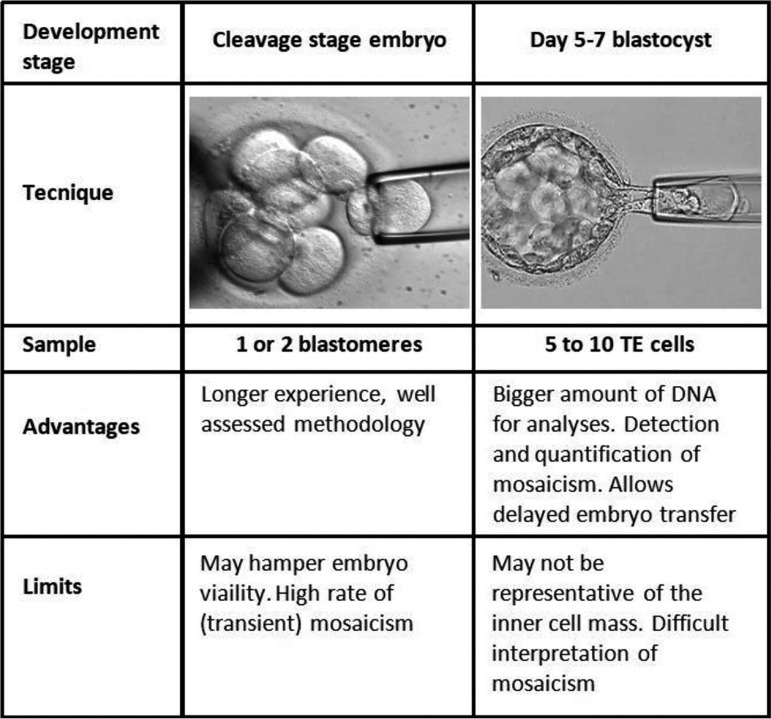
Preimplantation genetic testing for aneuploidy (PGT-A) and preimplantation genetic testing for monogenic diseases (PGT-M). Cleavage stage and trophectoderm biopsy. Adapted with permission from Sciorio & Dattilo (2020).

In this opinion paper, we would like to highlight the raised complexity of duties performed nowadays in a modern ART laboratory and to illustrate how those activities are correlated to additional time requirements for the completion of an IVF treatment in safety and providing quality service for the couple. As such, safe and efficient operation of the ART laboratory has become increasingly complicated, along with multiple responsibilities associated with proficiency and documentation. As reported by Alikani *et al*. (2014) in average in the 1980s about 9 hours were required to complete a cycle while currently it needs an average almost the double time. In particular, if the cycle requires performing embryo biopsy for preimplantation genetic assessment (Figure 2), the time needed will increase to more than 20 person hours (Alikani *et al*., 2014). Nowadays, an ART cycles take longer because they involve more complex technologies with suggested laboratory witnessing requirements, therefore, the number of embryologists needed to complete the daily duties, is considered to be increased as well. This number is correlated not only to the number of cycles annually performed, but also on the types of procedures offered; higher is this number and more personnel is required (Alikani *et al*., 2014; Practice Committees of the American Society for Reproductive Medicine and the Society for Assisted Reproductive Technology, 2013). Accordingly, in some countries, there is a tendency to adopt one embryologist for every 100-150 IVF cycles annually. The only guidelines available on this issue are dated, and are the one for administrative directors and human resources in the ART laboratory published in 2008 by the ASRM (Practice Committee of American Society for Reproductive Medicine & Practice Committee of Society for Assisted Reproductive Technology), which suggest two embryologists for up to 150 cycles annually, and 4 persons if the activities increased up to 600 cycles (Table 1). It is worth to mention how it is critical in the embryology laboratory the witness procedure, which in some countries is still considered an optional. An appropriate reproductive sample identification is important to remove the risk of gamete and embryo mismatches. Labeling all tubes and dishes containing gametes and embryos and employing manual double witnessing or electronic witnessing protocols, clearly decreases the risk of sample mismatching due to human error. Witness nowadays can be performed automatically or traditionally by a person (Forte *et al*., 2016). We do believe that witnessing procedure demands and ensure safety, it must be applied always at any single steps of an ART cycle, therefore a strict minimum of two people must be in the embryology laboratory at any time when clinical activities are carried out (Forte *et al*., 2016; Dyer, 2004). A witness can be anyone trained to do that process, even though very often it is another embryologist. Some units enforced trained nurses or laboratory assistants, or just personnel specifically hired for the purpose of witnessing at the weekend to reduce at the minimum the embryology staff (Novo *et al*., 2014).

**Table 1:** Revised from ASRM 2008.

Embryology staffing requirements in ART laboratory	
0 to 150 cycles per year	Minimun 2 embryologists
150 to 300 cycles per year	3 embryologists
300 to 600 cycles per year	4 embryologists
More than 600 cycles per year	One additional embryologist per 200 cycles

## THE BENEFIT OF TEAMWORK

Together with the evolution from research towards worldwide routine application, ART is confronted with increasing regulatory requirements and professional standards for embryology laboratories. In the beginning of this century both United States (US) and European authorities issued regulations to ensure quality and safety of human tissues and cells and now the European Union Tissues & Cells Directive 2004/23/EC (EUTCD) is implemented in all EU member states (Directive 2004/23/EC of the European Parliament and of the Council of 31 March 2004). It is now required to implement a Quality Management System (QMS) in an ART laboratory. Furthermore, embryology requires teamwork and the coordination of activities between the team is extremely important. Effective communication among the members of the laboratory is critical to decrease inter-observer variability. The main areas which require regular inspection are: instrument maintenance (including cryo-banks), the management of gametes and embryo banks in donation cycles, receiving and stocking of samples, embryo biopsy and preimplantation genetic assessment, as well as the shipping of the samples, communication concerning genetic test results and finally the management of disposable materials and culture media, including lot numbers and expiration dates. Teamwork is an important element in IVF laboratories to reduce risk of error (Jimena *et al*., 2016). It indicates an active process that involves the coordination and collaboration of each care team member. Choucair *et al.* (2021) stated that teamwork is a non-technical skill of key importance that contributes an embryologist’s success beside decision-making and stress management. The ART cycle process involves a series of strictly controlled events, with accurate attention to detail, performed by a team where everyone has a specific role to play (Choucair *et al*., 2021). The team is required to work carefully for long hours in environmentally controlled conditions, often without natural light. Incompetent or weak staffing numbers are often correlated with stress and might negatively influence the overall pregnancy outcome of the clinic (Mortimer *et al*., 2018).

## ADDITIONAL REMARKS AND CONCLUSIONS

**Figure 3:**
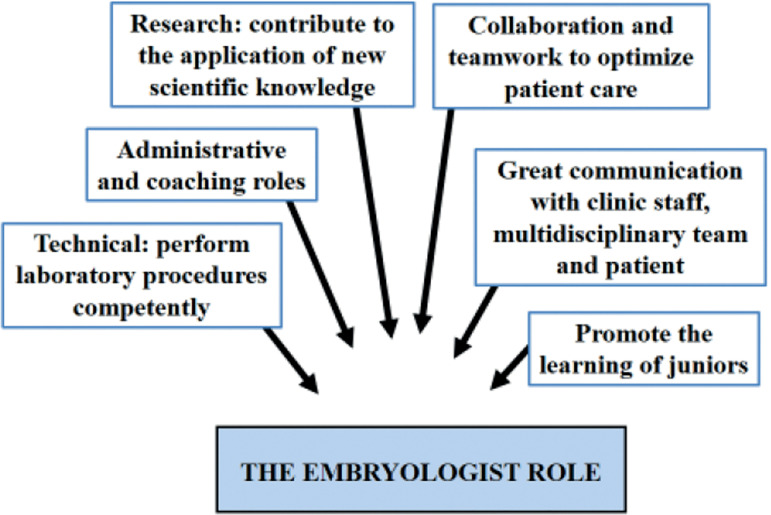
The key duties of the embryologist.

Embryologists are not only expected to use critical thinking skills for problem-solving and troubleshooting, but they also need to be aware of, and work conform to, the ethical and legal issues related to ART including Quality Management systems requirements. Furthermore, although guidelines advice that safe and efficient ART laboratory operation necessitates one embryologist for every 100-150 MAR treatments per year, appriases suggest that this falls short of the average recommended staffing, introducing further risks. It is probably mandatory to implement strict workloads, reducing each laboratory staff member’s hours to include work breaks. Every clinic should check its staff numbers, work volume, and ratio of senior to junior embryologists to determine appropriate staffing (Alpha Scientists in Reproductive Medicine, 2015; Practice Committee of American Society for Reproductive Medicine & Practice Committee of Society for Assisted Reproductive Technology, 2008; McCulloh, 2012). The role of the clinical embryologist has changed profoundly over time (Figure 3). The embryologist has always been considered a highly skilled occupation, widely trained to perform sensitive procedures where the margin for error is close to zero. IVF administrators should understand of the raised staff time requirements for some tasks that despite have been around for decades, they have seen a substantial increment in time-consuming over the years, including extended culture to blastocyst, freeze-all cycles, vitrification-warming, time-lapse annotations, monitoring to blastocyst stage and preimplantation genetic testing. Additionally, manipulating human gametes and embryo every day involves serious risk of errors, especially when the operator is mentally exhausted or working under pressure. Mental exhaustion leads to loss of focus, loss of attention and might cause disinterest, as well as reduced productivity. Embryologists are expected to use critical intelligent and competence to solve problems and to work in comply with the ethical and legal issues related to MAR treatment. Some qualities needed from an embryologist include: manual ability and precision, visual-movement coordination, calm and speedily in performing procedures, attention to detail, good judgement, rapid decision making and the capacity to work under stressful conditions. As well as personal qualities including a strong work ethic, integrity and trust also represent key features of effective embryologists. Albeit the efficiency should improve when more procedures are performed, it need to be mentioned that embryologists are faced with increasing responsibility, and therefore in case of shortage of embryologist staff, it might increase the risk of errors. To conclude, the current opinion paper on ART activities should encourage innovative guidelines from the body regulators on the embryology staffing that better reflect both the new technologies and processes performed in the modern IVF laboratory, in order to assure a safety and successful MAR treatment for patients.
